# SOCIAL ISOLATION AND OLDER ADULTS: UNDERSTANDING THEIR EXPERIENCE

**DOI:** 10.1093/geroni/igz038.1346

**Published:** 2019-11-08

**Authors:** Bonnie Jeffery, Tom McIntosh, Nuelle Novik

**Affiliations:** University of Regina, Regina, Saskatchewan, Canada

## Abstract

This presentation will focus on a unique undertaking where three provincial organizations in Saskatchewan, Canada implemented nine projects to address social isolation for rural and urban older adults across a geography that encompasses one-half of the province. A survey of older adults was conducted to assess their level of social isolation in order to gain a more thorough understanding of the experiences of social isolation among community dwelling older adults. Key variables of interest included: older adult access to services and supports, participation in activities, feelings of being valued by others, barriers to supports and services, and the overall isolation experienced by older adults. The responses from 1,719 urban and rural older adults indicate that 24.1% of respondents felt that they lack support, 17.2% feel less connected to family and friends, and 16.8% of respondents do not feel valued by their friends and family. Overall, almost one-quarter (23.9%) of the survey respondents score ‘high’ or ‘medium’ on a Social Isolation Index. One-third of respondents report they experience barriers to participation in activities outside the home. Several key categories of barriers were identified: health, personal, environmental, social, transportation and systemic. Respondents identified accommodation, services, practices, and activities as areas where their community could assist in participation of community activities outside of the home. Social isolation can have serious health consequences for older adults. The results of this survey highlight several key areas that older adults identify as important for reducing their feelings of isolation and enhancing their overall health and well-being.

